# Evaluation of Immunosuppressive Therapy Use for Tracheal Transplantation with Trachea-Mimetic Bellows Scaffolds in a Rabbit Model

**DOI:** 10.1155/2017/5205476

**Published:** 2017-10-26

**Authors:** Jae Yeon Lee, Jeong Hun Park, Soo Jin Son, Mina Han, Gonhyung Kim, Seong Soo Kang, Seok Hwa Choi, Dong-Woo Cho

**Affiliations:** ^1^Department of Mechanical Engineering, POSTECH, Pohang 790-784, Republic of Korea; ^2^Veterinary Medical Center, Chungbuk National University, Cheongju 361-763, Republic of Korea; ^3^College of Veterinary Medicine, Chonnam National University, Gwangju 500-757, Republic of Korea

## Abstract

The objective of this study was to evaluate the use of immunosuppressive therapy with high-dose cyclosporine, high-dose azathioprine, and a combination of low-dose cyclosporine and azathioprine after tracheal reconstruction by using a trachea-mimetic graft of polycaprolactone (PCL) bellows-type scaffold in a rabbit model. Twenty-four healthy New Zealand white rabbits were used in the study. All underwent circumferential tracheal replacement using tissue-engineered tracheal graft, prepared from PCL bellows scaffold reinforced with silicone ring, collagen hydrogel, and human turbinate mesenchymal stromal cell (hTMSC) sheets. The control group (Group 1) received no medication. The three experimental groups were given daily cyclosporine intramuscular doses of 10 mg/kg (Group 2), azathioprine oral doses of 5 mg/kg (Group 3), and azathioprine oral doses of 2.5 mg/kg plus cyclosporine intramuscular doses of 5 mg/kg (Group 4) for 4 weeks or until death. Group 1 had longer survival times compared to Group 2 or Group 3. Each group except for Group 1 experienced decreases in amount of nutrition and weight loss. In addition, compared with the other groups, Group 2 had significantly increased serum interleukin-2 and interferon-*γ* levels 7 days after transplantation. The results of this study showed that the administration of cyclosporine and/or azathioprine after tracheal transplantation had no beneficial effects. Furthermore, the administration of cyclosporine had side effects, including extreme weight loss, respiratory distress, and diarrhea. Therefore, cyclosporine and azathioprine avoidance may be recommended for tracheal reconstruction using a native trachea-mimetic graft of PCL bellows-type scaffold in a rabbit model.

## 1. Introduction

A large-ranging tracheal resection is frequently required in patients with tumours involving the trachea or with benign, congenital, or inflammatory tracheal stenosis [1–6]. Some surgical procedures—such as resection with primary anastomosis, slide-tracheoplasty, and costal cartilage tracheoplasty—have been performed, but they have not allowed for the reconstruction of more extensive lesions [7–9].

Various tracheal substitutes and various techniques of reconstruction have been investigated, including an artificial trachea scaffold, autogenous tissue, and an allotransplant, but resection remains a clinically unsolved surgical and biological problem. Studies on prosthetic grafts have been reported, with unsatisfactory results [10–14]. The major obstacles to successful clinical transplantation of the trachea have been the immunogenicity of the tracheal wall and the restoration of its blood supply.

An adequate level of immunosuppression is required for preventing the host's immune system from rejecting the transplanted organ and for improvements in outcome after transplantation [15, 16]. Although many immunosuppressants have become available or are being investigated for clinical use, none of them is perfect because of their adverse effects. To achieve maximum therapeutic effect with minimum toxicity, two or more immunosuppressants are most often used in combination. Although azathioprine-and-cyclosporine combination immunosuppressive therapy is the standard immunosuppressive protocol for organ transplantation surgery [17], there are few studies about the effects of those immunosuppressants in rabbit models. The white New Zealand rabbit* (Oryctolagus cuniculus)* is frequently used in a variety of experiments, including organ transplantation surgery. However, information on the therapeutic effects of immunosuppressants on that animal as an experimental model is limited.

The objective of this study was to evaluate immunosuppressive therapy with high-dose cyclosporine, high-dose azathioprine, and a combination of low-dose cyclosporine and azathioprine after tracheal reconstruction using a native trachea-mimetic graft of polycaprolactone (PCL) bellows-type scaffold in a rabbit model.

## 2. Methods

### 2.1. Animals and Study Design

Twenty-four healthy New Zealand white rabbits weighing 2.5 to 3.5 kg were included in this study. The protocol for the experiment was approved by the Laboratory Animal Research Center of Chungbuk National University (Approval Number CBNUA-714-14-01). All rabbits were housed in individual cages throughout the experimental period. Water and food were supplied ad libitum. All animals underwent tracheal transplantation using PCL bellows-type scaffold. After the transplantations, rabbits were randomly assigned into four groups of 6 each.

A control group (group 1) received no medication. The three experimental groups were administered daily cyclosporine (CIPOL INJ®; CKDpharm, Seoul) intramuscular doses of 10 mg/kg (Group 2), azathioprine (Immuthera Tab®; Celltrion Pharm, Seoul) oral doses of 5 mg/kg (Group 3), and azathioprine oral doses of 2.5 mg/kg plus cyclosporine intramuscular doses of 5 mg/kg (Group 4) for 4 weeks or until death. All animals were carefully monitored during the 28 days of observation for anastomotic complications or infection. At 28 days, the animals were sacrificed following euthanasia guidelines adapted from the American Veterinary Medical Association Guidelines for the Euthanasia of Animals.

### 2.2. Preparation of PCL Bellows-Type Scaffold Fabrication

A scaffold for tracheal graft was developed as a bellows structure with a native trachea-mimetic design. PCL (molecular weight 70,000–100,000, Wako Pure Chemical Industries, Osaka, Japan) was used to fabricate the framework of the tracheal scaffold. The PCL solution was prepared by dissolving PCL grains in chloroform (150% w/v) and injected into the sacrificial mould made of alkali-soluble photopolymer. The sacrificial mould filled with PCL solution was immersed in isopropyl alcohol to harden the PCL by extracting the chloroform for 24 h. Subsequently, the sacrificial mould with PCL was immersed in a 0.5 N NaOH solution, and gentle stirring for 8 h was conducted to selectively dissolve the sacrificial mould. After sacrificial mould removal, the bellows framework that remained was washed with distilled water. The framework was a fine PCL bellows structure 12 mm long, with an internal diameter of 5 mm, with pore size of 200 *μ*m and wall thickness of 300 *μ*m. The framework was then immersed in 1% collagen solution (Nippon Meat Packers, Inc., Osaka, Japan) at 4°C for 3 h. The combination was subsequently dried at room temperature for 1 h. The collagen layer on the surface of the PCL framework was cross-linked using 1-ethyl-3-(3-dimethylaminopropyl) carbodiimide and N-hydroxysuccinimide. The process was repeated 3 times. The silicone (SILASTIC®, Dow corning corporation, USA) ring layer on the surface of the PCL framework was created using sterilized syringe. Human turbinate mesenchymal stromal cell (hTMSC) sheets were then transferred onto the collagen gel layer of the PCL graft using sterilized paper membrane. Completed hTMSC sheets graft was cultured in vitro for 3 days before implantation.

### 2.3. Surgical Procedure

The rabbits were anesthetized with a cocktail of 35 mg/kg ketamine (ketamine 50 Inj., Yuhanyanhang Co., Seoul) and 5 mg/kg xylazine (Rompun Inj., Bayer Korea, Seoul) administered intramuscularly. Prior to surgery, the skin was shaved and then cleaned with a mixture of iodine and 70% ethanol. The skin and subcutaneous tissue were incised at the midline from 1 cm below the larynx to a length of 6 cm. The cervical trachea was exposed by midline separation of the paired strap muscles and mobilized circumferentially for 3–6 cm according to the planned resection. Two centimetres of the trachea was resected, and the prosthesis and the cut ends of the trachea were end-to-end anastomosed by simple interrupted sutures with 5-0 Maxon (Covidien). In all groups, fibrin glue (ISSEEL™ Kit, Baxter India, Haryana, India) was applied over the anastomosis and surgical field. All animals were administered intramuscularly with antibiotic (amoxicillin 20 mg/kg, Foxolin Inj., Samjin Pharm, Seoul) and analgesic (tramadol 3 mg/kg, tramadol Inj., Dongkwang Pharm, Seoul) for 7 days.

### 2.4. Clinical Evaluation

The rabbits were observed daily for coughing and dyspnoea. Coughing was recorded as absent, mild, moderate, or severe. At the same time, body weight, respiratory distress, and diarrhea were also examined. Scoring of clinical symptoms and signs including anorexia, diarrhea, and coughing was divided into 4 categories depending on the severity: absent (0), light (1), moderate (2), and severe (3). The cervical incisions were evaluated daily for swelling, inflammation, seroma formation, and subcutaneous emphysema. The rabbits' graft survival times were calculated for each group from operation day to 28 days or until death.

### 2.5. Magnetic Resonance Imaging

Magnetic resonance imaging (MRI) was performed in all animals just prior to sacrifice at 28 days; the degree of inflammation, changes in diameter, and oedema of transplanted trachea and adjacent organs were evaluated. MRI was performed using a 1.5-Tesla superconducting MR system (Siemens Medical Solutions, Erlangen, Germany) and a phase-array body coil with four active segments.

### 2.6. Quantification of Interleukin-2 and Interferon-*γ*

On day 7 after transplantations, peripheral blood was collected from the ear vein. Concentrations of interleukin-2 and interferon-*γ* were measured by means of enzyme-linked immunosorbent assay with Quantikine rabbit immunoassay kits (Rabbit Interleukin-2 and Rabbit Interferon-*γ* ELISA Kit, Cusabio Biotech Co., Ltd., Muhan, China) according to the manufacturer's instructions.

### 2.7. Histological Examination

Histological assessments were performed to evaluate the regenerative status of the operated site, the tracheal structure, vascular density, and inflammatory cell infiltration at 4 weeks postoperatively or at death. Tissues were fixed in 10% formalin at 4°C for more than 24 h and embedded in paraffin for histological evaluation. Tissue blocks embedded in paraffin were serially cut for 4-*μ*m thick sections and stained with hematoxylin, eosin, and Alcian Blue. Each section was observed under a light microscope (BX50, Olympus, Tokyo).

### 2.8. Statistical Analysis

Values were expressed as means and standard deviation. Kruskal–Wallis analysis of variance was used for group comparisons. Between-group differences were compared by a Mann–Whitney *U* test. The Friedman and Wilcoxon tests were used for within-group data analysis. The level of statistical significance was set at *p* < 0.05. All statistics were analysed with SPSS software ver. 19.0 (SPSS, Chicago, USA).

## 3. Results

### 3.1. Graft Survival

**Figure 1:**
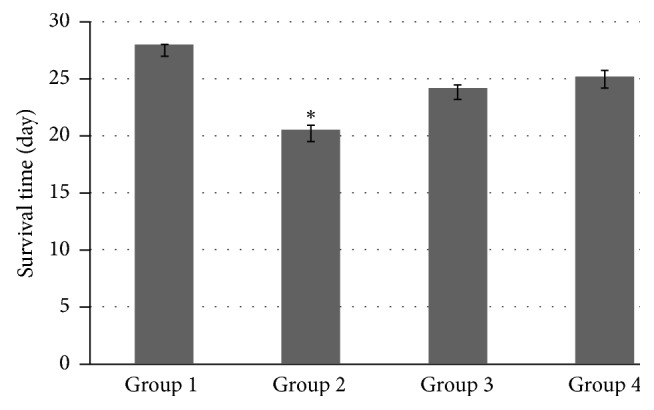
The untreated group had longer survival time than either cyclosporine group or azathioprine group. Group 1: no medication, Group 2: 10 mg/kg cyclosporine, Group 3: 5 mg/kg azathioprine, and Group 4: 2.5 mg/kg azathioprine plus 5 mg/kg cyclosporine. ^*∗*^Significantly different (*p* < 0.05) among groups.

Graft survival time is shown in Figure 1. In Group 1, the median survival time of the grafts was 28 days in tracheal transplantation. The median survival time of the grafts in Groups 2, 3, and 4 was 20.5 ± 2.38 days, 24.2 ± 2.60 days, and 25.2 ± 5.50 days, respectively. Group 1 had longer survival compared to Group 2 or 3.

### 3.2. Clinical Evaluation

Clinical assessment is shown in Table 1. Respiratory distress such as any kind of breathing-difficulty sound or symptoms was not observed in Group 1. In Group 2, all rabbits had severe respiratory distress such as inspiratory sound or stridor. Two rabbits in Group 4 and 3 rabbits in Group 3 developed mild respiratory distress such as shortness of breath, snoring, and goose-honking sounds. The remaining 3 rabbits in Group 3 had moderate degrees of respiratory distress similar to wheezing. In addition, 4 rabbits in group 2 had severe respiratory distress and watery faeces or diarrhea. Weight loss and poor appetite were also confirmed in some animals. Each group except for Group 1 experienced decreases in amount of nutrition and weight loss. Four rabbits in Group 2 and 2 rabbits in Group 4 experienced extreme weight loss to the extent of 48% because of respiratory distress and/or gastrointestinal signs such as diarrhea.

### 3.3. Magnetic Resonance Imaging

**Figure 2:**
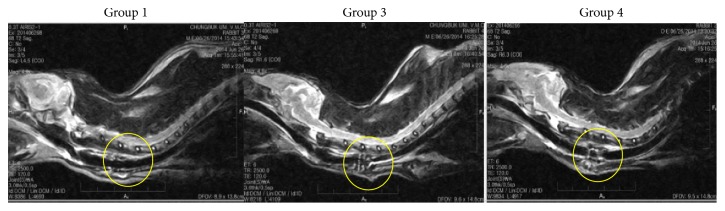
MRI appearance of the scaffold implanted into the cervical trachea of rabbit 28 days after the operation in Groups 1, 3, and 4. MRI shows the degree of inflammation and changes in diameter and edema of transplanted trachea and adjacent organs as conducted on rabbits in each group 28 days after implantation (yellow circle). Group 1: no medication, Group 2: 10 mg/kg cyclosporine, Group 3: 5 mg/kg azathioprine, and Group 4: 2.5 mg/kg azathioprine plus 5 mg/kg cyclosporine.

Magnetic resonance imaging shows the degree of inflammation as well as changes in diameter and oedema of transplanted trachea and adjacent organs as conducted on rabbits in each group 28 days after implantation (Figure 2). All rabbits in Group 2 died before MRI examination (data for Group 2 is not shown). In Group 1, the implanted prostheses were incorporated with the native trachea at the interfaces, but, in the other groups, there were morbid tissue reactions between adjacent organs, including blood vessels and fluids. In Groups 3 and 4, the irregular margin of surrounding tissues, swelling, and inflammation were confirmed; and tracheal lumens were significantly narrower with the mixture of inflammatory exudates compared with Group 1.

### 3.4. Serum Interleukin-2 and Interferon-*γ* Levels

**Figure 3:**
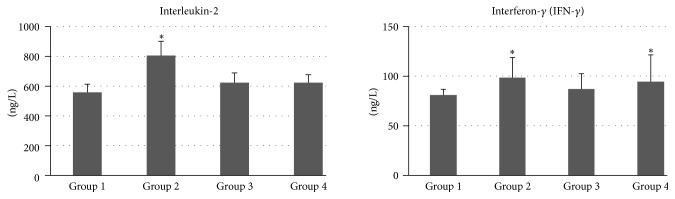
The levels of IL-2 and IFN-*γ* in peripheral blood. On day 7 after transplantation, the level of serum IL-2 and IFN-*γ* in group 2 was higher than that of other groups. Group 1: no medication, Group 2: 10 mg/kg cyclosporine, Group 3: 5 mg/kg azathioprine, and Group 4: 2.5 mg/kg azathioprine plus 5 mg/kg cyclosporine. ^*∗*^Significantly different (*p* < 0.05) among groups.

On day 7 after transplantation, levels of interleukin-2 in Groups 1, 2, 3, and 4 were 558.25 ± 50.40 ng/L, 804.25 ± 93.13 ng/L, 622.25 ± 67.20 ng/L, and 645.80 ± 49.89 ng/L, respectively (Figure 3). The level of interleukin-2 in Group 1 was lower than that of Groups 2 and 3. The levels of interferon-*γ* in Groups 1, 2, 3, and 4 were 82.40 ± 4.96 ng/L, 99.25 ± 20.36 ng/L, 88.00 ± 14.67 ng/L, and 95.25 ± 26.09 ng/L, respectively (Figure 3). Compared with Groups 1, 3, and 4, Group 2 had significantly increased serum interleukin-2 and interferon-*γ* levels 7 days after transplantation.

### 3.5. Histopathological Findings

**Figure 4:**
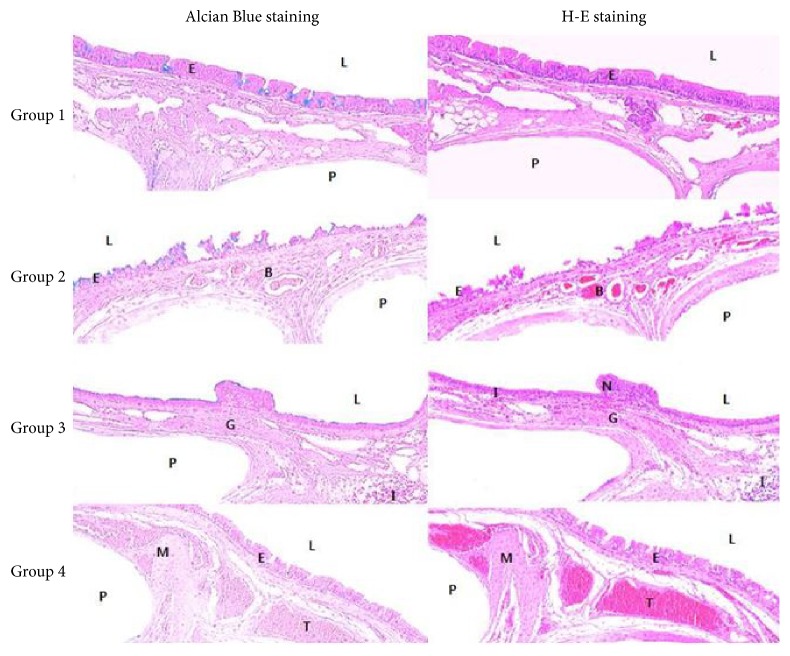
Histopathological view of the tracheal graft site at the completion of the study (day 28 or death time). At day 28 after operation, the regenerated neomucosa with an epithelial lining is observed on the scaffold and tracheal luminal surface was completely covered with epithelial cells in Group 1. Marked fibroblastic proliferation, mononuclear cell infiltration, high vessel density, and epithelial thickness were observed in Groups 3 and 4. In Group 2, fibroproliferative tissue with fibrosis below the epithelium is less evident. Histopathologic examination revealed nonepithelialized epithelium (Alcian Blue and hematoxylin and eosin staining; original magnification, ×40). Group 1: no medication, Group 2: 10 mg/kg cyclosporine, Group 3: 5 mg/kg azathioprine, and Group 4: 2.5 mg/kg azathioprine plus 5 mg/kg cyclosporine. N: nonciliated cell, M: hypertrophy of mucosal smooth muscle, I: inflammatory cell, G: hyperplasia of submucosal mucous gland, L: tracheal lumen, B: blood vessels, T: trachealis muscle, E: epithelium, and P: polycaprolactone (PCL) bellows-type scaffold.

Histopathological examination revealed that Group 1 showed complete reepithelialization in the subepithelial area, without any inflammation (Figure 4). Histologically, marked fibroblastic proliferation, mononuclear cell infiltration, high vessel density, and epithelial thickness were observed in Groups 2, 3 and 4. In Group 2, fibroproliferative tissue with fibrosis below the epithelium was less evident. Histopathological examination revealed nonepithelialized epithelium.

## 4. Discussion

The aim of this study was to evaluate the immunosuppressive effect of the use of cyclosporine and azathioprine in the prevention of acute graft rejection. The hypothesis was based on the different mechanisms of action and the different side effects of cyclosporine and azathioprine.

The result of this study showed the longest survival time and least number of harmful effects in Group 1. Compared with Group 1, cyclosporine and/or azathioprine administration induced severe adverse effects, including body weight loss, respiratory distress, and diarrhea.

The introduction of cyclosporine in clinical organ transplantation has improved the efficacy of immunosuppressive treatment, leading to a decline in the incidence of acute-rejection episodes and allograft loss in the first year after transplantation. However, cyclosporine has many toxic side effects such as exacerbation or genesis of hypertension, hyperlipidaemia, and nephrotoxicity [18].

Azathioprine is a purine analogue and has been used as an immunosuppressive agent to suppress the proliferation of B and T lymphocytes by interfering with normal purine synthesis and inhibiting DNA synthesis. The major side effects of azathioprine are bone marrow suppression and hepatotoxicity [19].

Recently, combinations of immunosuppressive agents have proved to be effective strategies to inhibit or prevent activity of the immune system, thereby facilitating decreases in each single drug's dosages and adverse effects. Currently, triple immunosuppressive therapy consisting of corticosteroids, azathioprine, and cyclosporine became the standard immunosuppressive protocol for organ transplantation [20]. However, the rabbit is a corticosteroid-sensitive species. Therefore, even small, one-time doses can cause severe changes in rabbits [21]. Moreover, compared with other animal models of organ transplantation, tracheal transplantation has not worked well in the rabbit model.

Transplantation medicine is one of the most difficult and complex areas of modern medicine. Transplant rejection is a major problem that can arise after any type of solid-organ transplantation, and certain treatments with completely novel mechanisms of action are now undergoing early clinical trials and should prove to be valuable additions to immunosuppressive therapy regimens. The use of immunosuppressive drugs and serotyping to determine the best donor-recipient match can reduce transplant rejection and improve transplant outcome [22].

In our present study, we evaluated combination immunomodulatory therapy with high-dose azathioprine, high-dose cyclosporine, and combination therapy with low-dose cyclosporine–azathioprine after tracheal reconstruction surgery by using a native trachea-mimetic graft of PCL bellows type. Our rabbit model showed the longest survival days and the least number of side effects in Group 1. In addition, any immunosuppressive agent for the avoidance of respiratory distress resulting from inflammation may not be effective and may even lead to severe adverse effects. Cyclosporine especially could cause side effects such as significantly decreased survival, extreme respiratory distress, and severe diarrhea, resulting in alarming weight loss; it could also result in death. More severe clinical signs were observed in Group 2 compared with Groups 1, 3, and 4. Therefore, it could cause more severe adverse effects in a rabbit model when the dose is not carefully selected in the case of short-term immunomodulatory therapy in rabbits. In addition, serum samples from rabbits treated with different immunosuppressants showed significant increases in interleukin-2 and interferon-*γ* levels in Group 2 compared with the other groups in our study. The increase in production of interleukin-2 and interferon-*γ* might reveal no immunosuppressive effects of cyclosporine in rabbits. Because both interleukin-2 and interferon-*γ* are rejection products in activated lymphocytes, both of them are good markers of pharmacodynamic immunosuppressive effects. Moreover, histopathological examination revealed nonepithelialized epithelium in Group 2.

In conclusion, the results of this study showed that the administration of cyclosporine and/or azathioprine after tracheal transplantation had no beneficial effects. Furthermore, the administration of cyclosporine had side effects including extreme weight loss, respiratory distress, and diarrhea. Therefore, cyclosporine and azathioprine avoidance may be recommended for tracheal reconstruction using a native trachea-mimetic graft of PCL bellows-type scaffold in a rabbit model.

## Figures and Tables

**Table 1:** Body weight and scores of clinical assessment in rabbits.

Group	Weight (kg)	Respiratory distress grade	Anorexia	Diarrhea
1	2.50 ± 0.05	0.0 ± 0.0	0.0 ± 0.0	0.0 ± 0.0
2	1.65 ± 0.45	2.5 ± 0.57^a,b^	2.25 ± 0.50^a,b,c^	2.25 ± 0.15^a,b,c^
3	2.25 ± 0.23	1.25 ± 0.95^a^	0.75 ± 0.50^a^	0.5 ± 0.57^a^
4	2.27 ± 0.51	2.50 ± 0.05^a^	1.0 ± 1.41^a^	0.75 ± 0.15^a^

Data are expressed as mean ± SD (*n* = 6). Group 1: no medication, Group 2: 10 mg/kg cyclosporine, Group 3: 5 mg/kg azathioprine, and Group 4: 2.5 mg/kg azathioprine plus 5 mg/kg cyclosporine. ^a^Significantly different (*p* < 0.05) from Group 1. ^b^Significantly different (*p* < 0.05) from Group 3. ^c^Significantly different (*p* < 0.05) from Group 4.
